# Assessment of the uptake of neonatal and young infant referrals by community health workers to public health facilities in an urban informal settlement, KwaZulu-Natal, South Africa

**DOI:** 10.1186/1472-6963-13-47

**Published:** 2013-02-06

**Authors:** Duduzile Nsibande, Tanya Doherty, Petrida Ijumba, Mark Tomlinson, Debra Jackson, David Sanders, Joy Lawn

**Affiliations:** 1 Health Systems Research Unit, Medical Research Council, 491 Ridge Road, Durban, South Africa; 2 School of Public Health, University of the Western Cape, Bellville, Cape Town, South Africa; 3 Department of Psychology, University of Stellenbosch, Stellenbosch, South Africa; 4 Saving Newborn Lives, Save the Children, Cape Town, South Africa; 5 MARCH center, London School of Hygiene and Tropical Medicine, London, UK

**Keywords:** Health seeking behaviour, Child health, Neonate, Newborn, Community health workers, Referral completion, Primary health care, Urban health, HIV

## Abstract

**Background:**

Globally, 40% of the 7.6 million deaths of children under five every year occur in the neonatal period (first 28 days after birth). Increased and earlier recognition of illness facilitated by community health workers (CHWs), coupled with effective referral systems can result in better child health outcomes. This model has not been tested in a peri-urban poor setting in Africa, or in a high HIV context.

**Methods:**

The Good Start Saving Newborn Lives (SNL) study (ISRCTN41046462) conducted in Umlazi, KwaZulu-Natal, was a community randomized trial to assess the effect of an integrated home visit package delivered to mothers by CHWs during pregnancy and post-delivery on uptake of PMTCT interventions and appropriate newborn care practices. CHWs were trained to refer babies with illnesses or identified danger signs. The aim of this sub-study was to assess the effectiveness of this referral system by describing CHW referral completion rates as well as mothers’ health-care seeking practices. Interviews were conducted using a structured questionnaire with all mothers whose babies had been referred by a CHW since the start of the SNL trial. Descriptive analysis was conducted to describe referral completion and health seeking behaviour of mothers.

**Results:**

Of the 2423 women enrolled in the SNL study, 148 sick infants were referred between June 2008 and June 2010. 62% of referrals occurred during the first 4 weeks of life and 22% between birth and 2 weeks of age. Almost all mothers (95%) completed the referral as advised by CHWs. Difficulty breathing, rash and redness/discharge around the cord accounted for the highest number of referrals (26%, 19% and 17% respectively). Only16% of health workers gave written feedback on the outcome of the referral to the referring CHW.

**Conclusions:**

We found high compliance with CHW referral of sick babies in an urban South African township. This suggests that CHWs can play a significant role, within community outreach teams, to improve newborn health and reduce child mortality. This supports the current primary health care re-engineering process being undertaken by the South African National Department of Health which involves the establishment of family health worker teams including CHWs.

**Trial registration number:**

ISRCTN41046462

## Background

South Africa is one of the ten countries (from a total of 193) that have shown a reversal in progress for the Millennium Development Goal (MDG) 5 to reduce under-5 mortality rates by 2015 [1]. HIV/AIDS has been identified as one of the leading contributors to this increase, estimated to account for over half of under-five mortality [2]. Nearly 9 children die every hour in South Africa from preventable causes, with one in three child deaths being due to neonatal causes [3].

Across South Africa many childhood deaths occur outside the health system, and at least 25% of those included in mortality audits were related to delayed recognition of illness or late arrival at facilities. Of those deaths that occurred in facilities, over half were related to poor care or care received too late [2,4]. Home care and support is crucial; newborn babies are especially vulnerable if they are ill and can die quickly, literally within hours. Hence recognition by the mother or other caregivers and rapid reaction are critical to reducing deaths [5].

The South African government introduced and implemented policies based on primary health care (PHC) in 1994, to ensure equitable, accessible and affordable health care for all. These policies included the revitalisation of PHC facilities and introduction of the Free Health Care policy for pregnant women and children below the age of six [6]. In 2002, the Integrated Management of Childhood Illness (IMCI) programme was introduced in South Africa. However, establishing formal links between communities and health facilities has been problematic [7,8]. Non-functional referral strategies, constrained health systems, poor community health worker (CHW) training and supervision and lack of integration have been reported as major obstacles to effective PHC delivery [9].

Several studies have documented low referral completion rates of between 24 and 58% for neonates and children to first level hospitals but very few studies have focused on the effectiveness of community based referrals using CHWs [10-12]. The main objective of this paper is to report on the uptake of CHW referrals within a peri-urban settlement, to describe the referral process and health seeking behaviour of mothers.

## Methods

This referral study was a sub-study of a cluster randomized controlled trial known as Good Start Saving Newborn Lives (SNL) which was implemented in KwaZulu-Natal province between 2008 and 2011 (ISRCTN41046462). The goal of the trial was to develop, evaluate and cost an integrated and scalable home visit package delivered by CHWs, targeting pregnant and postnatal women and their newborns, to provide essential maternal/newborn care and support in accessing prevention of mother to child transmission of HIV (PMTCT). The study methods are described in detail elsewhere [13]. Briefly, the trial consisted of 30 randomized clusters (15 in each arm) within Umlazi township, a peri-urban sub-district near Durban city.

In the intervention arm, CHWs visited mother-baby pairs during the last trimester of pregnancy and during the early postnatal period to deliver a package of interventions relating to maternal and child health. Five post-natal home visits were conducted; within the first 24–48 hours, within day 3 or 4, within day 10–14, within 3–4 weeks, after 6 weeks, and with two extra visits for low birth weight newborns. One of the roles of CHWs was to counsel mothers on early identification of newborn danger signs and to refer to health care facilities where necessary to prevent complications. If danger signs were detected a CHW wrote a referral note to the local PHC clinic.

### Study design referral sub-study

For the referral sub-study primary data was collected from mothers who had received a referral from a CHW at any time during the period of the trial. The aim of this study was to provide information on compliance with CHW referrals to inform the planned national scale up of a CHW programme in South Africa.

### Study setting

Umlazi township has a population of approximately one million which is served by a district hospital and 10 PHC facilities. The housing settlements consist of a mixture of formal and informal housing. The estimated infant mortality rate for Kwazulu-Natal province is 56/1000 live births [14] and the neonatal mortality rate around 23/1000 live births [15]. The antenatal HIV prevalence for the district was 38% in 2011 [16]. All PHC facilities offer a comprehensive PHC service and over 85% of all births in Umlazi occur in one major public hospital, Prince Mshiyeni Memorial Hospital (PMMH).

### Study participants

All women listed in CHW referral books as participating in the SNL study, whose babies had been referred to health facilities were invited to participate in this sub-study. Mothers who no longer resided in Umlazi during the time of the referral data collection, and those who denied receiving referrals despite being listed in CHW referral books were excluded from this sub- study.

### Data collection

A pre-coded structured questionnaire was developed. Major sections contained demographic information, health-seeking behaviour, questions related to the referral process, perceptions of quality of care and a review of the Road to Health Chart (RTHC). Most questions related to the mothers’ experiences during the last CHW referral of the infant. The questionnaire was translated into the local language and loaded onto a cell phone running a survey software package [17] for electronic data collection in the field [18]. Between January and June 2010 an independent data collector, who was not involved in the main SNL trial conducted interviews with mothers.

### Data management and analysis

Data were converted from the mobile researcher database to excel and imported to STATA version 11 and Epi Info for analysis. Textual responses, mostly from ‘other’ categories in questions, were grouped into common themes, coded and then entered into Microsoft Excel. Data were explored initially using basic frequencies for categorical data and means for continuous data. Comparative analysis was undertaken using chi square tests for categorical data and one way analysis of variance for continuous data for mothers that completed and those that did not complete referrals. A significance level of 0.05 was used.

### Ethical considerations

Ethical approval for the referral sub-study was obtained from the University of the Western Cape Ethics Committee and permission was obtained from the KwaZulu-Natal Provincial Department of Health. Written informed consent was obtained from willing participants.

## Results

### Characteristics of mothers and infants

Characteristics of mothers are shown in Table 1. The mean age of mothers was 24 years. Most had attended some high school; the majority were single and unemployed. About half of mothers had piped water in their dwelling. From hospital records 39% of mothers were HIV positive, 56% negative and 5 (4%) had missing status. All interviews were conducted with the infants’ mothers, except one with a grandmother. The majority (71%) of infants were older than 16 weeks at the time of the interview. Less than half were still being breastfed with the lowest proportion amongst infants older than 16 weeks (37%) (Table 2). Six infants were reported to have died, with difficulty breathing being the major cause of death (83%). The mean age at the time of death was 4.2 months (range 2.1 - 7.3). All the infants who died had completed referrals, and all except one died in hospital. Deaths occurred at least one month after the CHW referrals. It is not known whether their deaths were related to the initial referrals by CHWs. 

**Table 1:** Characteristics of mothers who completed and did not complete referral

	**Referrals completed (n = 104)**	**Referral not completed (n = 6)**
Age, mean years	24.7 (5.9)	23.5 (3.6)
Mothers education, mean years	10.6 (1.7)	10.7 (2.5)
**Marital status**		
Single	85 (81.7)	5 (83.3)
Married	3 (2.9)	1 (16.7)
Co-habiting	10 (9.6)	0
Widowed	1 (0.9)	0
Missing	5 (4.8)	0
**Previous Children**		
Yes	56 (53.8)	4 (66.7)
No	43 (41.3)	2 (33.3)
Missing	5 (4.8)	0
**Employed**		
Part-time	8 (7.6)	0
Full time	2 (1.9)	1 (16.7)
Temporary	2 (1.9)	0
Not employed	87 (83.7)	5 (83.3)
Missing	5 (4.8)	0
**Child birth place**		
Public Hospital	90 (86.5)	6 (83.3)
Clinic/primary health centre	5 (4.8)	0
Private hospital	1 (0.9)	0
Other	3 (2.9)	1 (16.7)
Missing	5 (4.8)	0
**Electricity**		
Yes	95 (91.3)	6 (100)
No	4 (3.8)	0
Missing	5 (4.8)	0
**Cooking fuel**		
Wood	1 (0.9)	0
Paraffin/Kerosene	8 (7.7)	0
Electricity	90 (86.5)	6 (100)
Missing	5 (4.8)	0
**Water source**		
Piped Dwelling	54 (51.9)	3 (50)
Piped Yard	26 (25)	3 (50)
Piped Public	19 (18.2)	0
Missing	5 (4.8)	0
**HIV Positive**		
Yes	41 (39.4)	2 (33.3)
No	58 (55.8)	4 (66.6)
Missing	5 (4.8)	0

Data are number (%) or mean (Std Dev).

**Table 2:** Characteristics of infants of mothers who completed and did not complete referral

**Characteristics of infants**	**Referral completed (n = 104)**	**Referral not completed (n = 6)**
**Gender**		
Male	52 (50)	2 (33.3)
Female	52 (50)	4 (66.7)
**Age at interview**		
< 6weeks	8 (7.7)	1 (16.7)
6 weeks - 9 weeks	9 (8.9)	1 (16.7)
10 weeks −13 weeks	6 (5.7)	1 (16.7)
14 weeks −16 weeks	5 (4.8)	1 (16.7)
> 16 weeks	76 (73.0)	2 (33.3)
**Age at first referral**		
Less than 2weeks	22 (21.1)	2 (33.3)
2–4 weeks	41 (39.4)	3 (50)
5–8 weeks	32 (30.8)	0
9–12 weeks	7 (6.7)	1 (16.7)
Do not know	2 (1.9)	0
**Alive at time of interview**		
Alive	98 (94.2)	6 (100)
Died	6 (5.8)	0
**Breastfed in the 24 hours before the interview**		
Yes	38 (36.5)	4 (66.7)
No	60 (57.7)	2 (33.3)
Missing	6 (5.8)	0 (0)

Data are number (%).

### Referral completion

Out of 2423 mothers enrolled in the main SNL study a total of 148 (6%) were referred by a CHW at some point during the study. Of these 148, 10 mothers could not be contacted, 10 mothers denied having ever received infant referrals despite appearing in the referral records (these mothers were not interviewed), 16 mothers had relocated, 1 refused participation and 1 had died a week prior to the scheduled interview; hence it was not appropriate to interview the family so soon after the death. For the purpose of the study, we decided to exclude the 10 mothers who denied having been referred despite appearing in the CHW referral books. Interviews were conducted with 110 mothers who consented to participate; either in their homes or at the PMMH project office. The facility referral completion rate was 95% (104/110) for those mothers interviewed. If the infants who appeared in the referral log but whose mothers denied receiving a referral were included, and assumed to be non-completed referrals, then the referral completion rate would decrease to 87%. For the majority of infants [44/110] (40%) the first CHW referral was between the ages of 2–4 weeks, 29% (32/110) were between 5–8 weeks, 22% (24/110) were less than 2 weeks old and 7% (8/110) were between 9–12 weeks of age. Two mothers (2%) were unsure of the age (Table 2). Difficulty in breathing, rash and redness/discharge around the cord accounted for the highest number of referrals (26%, 19% and 17% respectively). Infants were referred to PHC facilities (92%), a public hospital (7%) and one mother consulted a general practitioner.

### Illness identification

There was a significant difference between mothers who completed the referral and those who did not with regard to recognition of danger signs. None of the six mothers who did not complete referral recognised any danger signs in their infants. Amongst completed referrals, about half of mothers (51%) were able to recognize danger signs that indicated that the child should be taken to the clinic or hospital (p = 0.01). The most common danger signs mothers identified were difficulty breathing (28%) and fever (24%).

### Referral delays

The time taken to complete referral was immediate (less than one hour) for a quarter of mothers (24%) but referral delays of up to 12 hours (12%) and beyond 12 hours (63%) were reported. The median number of days taken from receiving the referral from the CHW to attending a clinic was 1 day. Out of 79 women who delayed seeking care by more than an hour, 78 gave reasons for the delay. The most common reasons for delay reported by mothers were not realising the seriousness of the illness and the clinic being closed (Table 3). 

**Table 3:** Reasons for delay in referral completion (more than 1 hour)

	**n**	**%**
Did not realize seriousness	29	37.1
Clinic closed/too late to be attended	22	28.2
Other responsibilities or work	8	10.2
Lack of funds	6	7.6
Transportation not easy	3	3.9
Using other treatment	3	3.9
Fear of nurses attitudes	2	2.6
Mother ill	1	1.3
Weather	1	1.3
Traditional reasons	1	1.3
Facility too far	1	1.3
Don’t know	1	1.3
**Total**	78*	100

*1 mother did not give any reason for the delay.

Home treatment was used by 24% of mothers, the most common being over-the- counter medicines (58%), followed by eye instillation of breast milk for eye problems (23%). The median duration of using home treatment was 3 days (range 1–7). None of the mothers reported using herbal medicines.

### Access

Eighty-three percent of mothers did not experience any difficulty with transport to facilities as most mothers (56%) walked to referral facilities whilst 38% took a taxi. The median cost of transport to facilities was R10.00 ($1.25). Although a minority stated they had difficulty getting money, none reported that they could not get it.

### Perceived quality of care

The longest waiting time at a facility was reported to be more than 2 hours for 22% of mothers; however, 31% reported waiting less than 30 minutes. The majority of mothers who brought referral slips reported that they were attended to promptly (78%). However, some mothers were told to follow the queue (10%) and 7% were told there was nothing wrong with the baby. Only 16% of health workers gave written feedback on the outcome of the referral to the referring CHW. The majority of mothers felt that the quality of service at health care facilities was good (71%), although a greater proportion rated the quality of service by CHWs as good (98%).

### Referral outcome

Referred infants were diagnosed by health facility staff as having difficulty breathing (13%), redness or discharge around the cord (13%), rash (12%), jaundice (11%), eye discharge (9%) diarrhoea (6%), oral thrush (6%) cough (5%) and fever (4%). The remaining 11 diagnoses included difficulty sucking, vomiting, constipation and colic and for a further 11 infants the diagnosis was unknown. Among mothers who completed referral, the majority (82%) reported that their infants’ condition improved. Among referred cases, ten (9%) were further referred to a hospital.

Mothers who did not complete referral stated that they did not realize the seriousness of the condition (33%), felt no treatment was necessary (33%) or chose other treatments (33%).

### Review of Road to Health Cards (RTHC)

Out of 109 RTHCs that were reviewed, only 54 (50%) had the mothers’ HIV status indicated. Out of those whose HIV status was marked, forty-eight (89%) were HIV positive and therefore their infants were HIV exposed. PCR test results were recorded on 3 out of 48 RTHCs of exposed infants of which one was HIV positive. Ninety-seven percent, 92% and 88% of infants were up to date with their 6 weeks, 10 weeks and 14 weeks immunizations respectively.

## Discussion

To our knowledge this is the first study assessing the outcome of referrals by CHWs to PHC clinics in an urban township area in sub-Saharan Africa. Overall for 70% (104/148) of infants referred by CHWs the outcome of the referral could be confirmed. Amongst interviewed mothers, 95% of referrals of infants by CHWs during the first 12 weeks of life were completed, which is much higher than reported elsewhere in Africa [19,20] and South Asia [21].

Most referrals were for neonates (62% between birth and 4 weeks of age), a critical period for timely referral to prevent death. Furthermore, most referrals were for respiratory problems which are a major cause of neonatal death in low and middle income countries.

A number of factors could potentially have contributed to the high referral completion rate in our study: firstly, Umlazi health facilities are easily accessible, most within walking distance. A study in Zambia also found that proximity to health facilities contributed to improved referral completion [22]. Secondly, CHWs were carefully selected and resided within their communities and this enhanced trust, respect and communication with caretakers. Thirdly, the use of referral letters resulted in less waiting times for most mothers. Kallander et al. in a case series study on referral in home management of malaria in Western Uganda indicated that use of referral slips and “counter referral” slips could contribute to improved referral compliance [23]. Finally, intensive training and field supervision of CHWs with weekly contact sessions for mentoring might have increased CHW counselling and assessment skills. The finding of high referral completion within an urban township context is encouraging in light of the current national Department of Health plans to formalise CHW involvement in the health sector. This result is an indication that mothers do respect CHW judgement and advice.

This study found a referral rate of 6% amongst the study cohort which is lower than the generally expected rate of around 10 to 15%. The reason for this lower referral rate could be that this study captured CHW referrals during the first 10 weeks after birth only (CHWs conducted home visits during the first 10 weeks after birth). Another plausible reason could be that a newborn illness is an emergency; it is possible that the neonates were taken to health facilities before CHWs conducted a home visit and these were not included in the CHW referrals.

We found that most mothers delayed more than 12 hours before completing a referral following CHW advice. Even a few hours delay can be fatal, especially in the early weeks of a baby’s life. In South Africa child deaths are audited as part of the Child Health Identification Programme. A death audit at King Edward Hospital in Durban found that 25% of child deaths were due to modifiable factors such as delay in seeking care and failure to realize the severity of illness [4]. In our study mothers cited not recognising the seriousness of illness and closed clinics as reasons for delays in consulting. Delays of up to 7 days were reported in western Nepal, Cambodia and western Uganda in health care seeking, with lack of money commonly mentioned as a cause for delays [23-25]. In contrast to other studies [26,27] we found that transport was not a barrier to referral completion in a context where the majority of mothers could walk to a facility.

In our study all mothers who did not complete referral reported that they failed to identify danger signs. It is possible that according to mothers’ judgement, infant illnesses may not have been viewed as serious. A study in western Nepal found that maternal perceived severity of illness was an important factor influencing care-seeking behaviour [24]. A longitudinal community-based study on mothers’ health-care seeking in South West Ethiopia found that mothers adopted a wait-and see attitude, hoping symptoms would resolve on their own which delayed consulting [28].

We found that less than a quarter of health workers gave feedback to the CHW on the outcome of the referral. Similar results of lack of formal written feedback were reported by Siddiqi et al. in Pakistan who found that none of the higher level facilities sent feedback on the outcome of referrals back to first level care facilities [29]. Poor feedback to CHWs following referrals is cause for concern given that the Department of Health is proposing to develop family health teams of CHWs linked to health facilities.

Our study had several limitations. Selection bias might have been introduced due to the numbers of mothers who were lost to follow up due to relocation or denying receiving referrals despite being on the lists. Recall bias might have occurred as events under study occurred between 2 weeks and 18 months prior to the interview. To address this, data from participants interviewed more than six months following referral were analysed separately to assess the potential for recall bias before pooling all data. Since severe illness or hospitalisation of an infant is a fairly major family event we do not anticipate that recall bias posed a meaningful problem.

## Conclusions

Our study, although small, provides evidence that mothers’ decisions on health seeking behaviours for their infants can be positively influenced by a community-based care and outreach strategy within the existing health-care system. Unanticipated barriers such as care-givers’ perception of illness severity, clinic organization and opening times were also found to play an important role in health- care seeking decisions. The results of this study highlight the potentially important role played by CHWs in the early detection of newborn illness and appropriate response. Understanding the individual and operational barriers to referral completion by policy makers, health planners and providers has implications for informing the revitalisation of a PHC-led health system currently being planned or implemented in South Africa and many other developing countries.

## Competing interests

The authors declare that they have no competing interests.

## Authors’ contributions

DN conceived the research topic and formulated the methods with advice from TD and PI. The data were extracted by DN, and DN wrote the first draft of the paper. DN and TD undertook the analysis and all authors contributed to the interpretation of the data and reviewed and edited the manuscript for important intellectual content. The opinions expressed are those of the authors alone. All authors read and approved the final manuscript.

## Pre-publication history

The pre-publication history for this paper can be accessed here:

http://www.biomedcentral.com/1472-6963/13/47/prepub
